# Neuropilin-1 modulates TGFβ signaling to drive glioblastoma growth and recurrence after anti-angiogenic therapy

**DOI:** 10.1371/journal.pone.0185065

**Published:** 2017-09-22

**Authors:** Sam C. Kwiatkowski, Paola A. Guerrero, Shinya Hirota, Zhihua Chen, John E. Morales, Manish Aghi, Joseph H. McCarty

**Affiliations:** 1 Department of Neurosurgery, University of Texas M.D. Anderson Cancer Center, Houston, Texas, United States of America; 2 Department of Neurosurgery, University of California at San Francisco, San Francisco, California, United States of America; Istituto per la Ricerca e la Cura del Cancro di Candiolo, ITALY

## Abstract

Glioblastoma (GBM) is a rapidly progressive brain cancer that exploits the neural microenvironment, and particularly blood vessels, for selective growth and survival. Anti-angiogenic agents such as the vascular endothelial growth factor-A (VEGF-A) blocking antibody bevacizumab yield short-term benefits to patients due to blood vessel regression and stabilization of vascular permeability. However, tumor recurrence is common, and this is associated with acquired resistance to bevacizumab. The mechanisms that drive acquired resistance and tumor recurrence in response to anti-angiogenic therapy remain largely unknown. Here, we report that Neuropilin-1 (Nrp1) regulates GBM growth and invasion by balancing tumor cell responses to VEGF-A and transforming growth factor βs (TGFβs). Nrp1 is expressed in GBM cells where it promotes TGFβ receptor internalization and signaling via Smad transcription factors. GBM that recur after bevacizumab treatment show down-regulation of Nrp1 expression, indicating that altering the balance between VEGF-A and TGFβ signaling is one mechanism that promotes resistance to anti-angiogenic agents. Collectively, these data reveal that Nrp1 plays a critical role in balancing responsiveness to VEGF-A versus TGFβ to regulate GBM growth, progression, and recurrence after anti-vascular therapy.

## Introduction

GBM is a malignant brain cancer that is distinguished from lower grade tumors, in part, by the development of hallmark angiogenesis pathologies, including florid microvascular cell proliferation and breakdown of the intratumoral blood-brain barrier [1]. GBM cells are also highly invasive, and often utilize blood vessels and their extracellular matrix (ECM)-rich basement membranes to disperse throughout the brain [2]. Invasive GBM cells often escape surgical resection and invariably contribute to tumor recurrence. Various ECM and growth factor signaling pathways that promote pathological angiogenesis and perivascular GBM cell invasion have been identified, including components of the VEGF-A and TGFβ signaling pathways [3]. VEGF-A is a potent inducer of angiogenesis in GBM via activation of VEGFR1 and VEGFR2 receptors, leading to increased endothelial cell proliferation, sprouting and permeability [4]. Recent data also reveal that GBM cells express VEGFRs, which signal to suppress tumor cell invasion [5, 6]. Inhibition of VEGF-A signaling via the neutralizing antibody bevacizumab blocks angiogenesis and leads to short-term improvements in progression free survival [7]. However, tumors often recur due to acquired resistance to bevacizumab, and this recurrence is frequently associated with robust perivascular tumor cell invasion [8].

The TGFβ signaling pathway is a major driver of GBM pathogenesis [9]. For example, during progression from low-grade astrocytoma to GBM, tumor cells activate TGFβ receptor signaling to promote proliferation, although increased expression of other growth factors such as PDGFBB also drive proliferation [10]. TGFβ signaling in GBM cells is stimulated by deposition of latent-TGFβ ligands in the ECM and their activation by αvβ8 integrin and other pathways [11]. In addition, activated microglia express TGFβ2 in response to neuroinflammation in the GBM microenvironment, which further promotes malignant tumor cell behaviors and angiogenesis pathologies [12]. TGFβ signaling is mediated primarily by type I and type II transmembrane receptors, which possess serine/threonine kinase activities and signal via multiple intracellular effector proteins, including ‘canonical’ Smad transcription factors [13].

Nrp1 is a co-receptor for multiple secreted factors such as semaphorins [14], VEGF-A [15], HGF [16], Hedgehogs [17], EGF [18], and PDGFBB [19] and TGFβs [20]. Due to its numerous co-receptor roles, Nrp1 participates in pleiotropic functions in many organs in a cell type-selective manner to modulate proliferation, migration, and survival. A role for Nrp1 in TGFβ receptor signaling has been demonstrated in myofibroblasts, immune cells and breast cancer cells [21–23]. In addition, Nrp1 modulates endothelial cell growth and sprouting largely via the canonical TGFβ signaling pathway during developmental angiogenesis in the central nervous system [24, 25]. Targeting Nrp1 with neutralizing antibodies in cancer cells leads to diminished growth and invasion, although these effects are only partly linked to defects in VEGF-A signaling [26]. Functions for Nrp1 in modulating TGFβ signaling in GBM cells, and how the Nrp1-TGFβ signaling cascade impacts blood vessels in the GBM microenvironment, have not been extensively investigated.

In this study, we analyze the roles of Nrp1 in GBM cell growth and recurrence after anti-angiogenic therapy via activation of TGFβ signaling. Nrp1 expression increases during tumor progression from low-grade to GBM, and is expressed in GBM cell lines and patient samples. Nrp1 acts as a co-receptor with TGFβR2 to enhance TGFβ receptor signaling via Smad3. Additionally, we show that Nrp1 expression is diminished in GBM cells in tumors that acquire resistance to bevacizumab. Hence, understanding how Nrp1-dependent TGFβ signaling drives GBM development and progression will help in identifying new therapeutic targets to counter the deadly growth and invasive behaviors in GBM.

## Materials and methods

### Ethics statement

Approval for the use of human specimens was obtained from the Institutional Review Board (IRB) at the University of Texas MD Anderson Cancer Center. The IRB waived the requirement for informed consent for previously collected residual tissues from surgical procedures stripped of unique patient identifiers according to the Declaration of Helsinki guidelines. Archived human GBM samples collected before and after bevacizumab treatment were obtained through a protocol approved by the IRB at the University of California San Francisco. All animal procedures and experiments conducted in this study were reviewed and approved by the University of Texas MD Anderson Cancer Center Institutional Animal Care and Use Committee (IACUC).

### GBM cell lines and human tumor samples

LN229 and U87 GBM cell lines, as well as HEK 293T cells, were purchased from the American Type Culture Collection (Manassas, VA). GBM cell lines were cultured in DMEM (Mediatech; Manassas, VA) supplemented with 10% fetal bovine serum (Atlanta Biologicals; Lawrenceville, GA) and antibiotics. Nrp1-dpendent LN229 cell proliferation was quantified by counting adherent cells grown in complete media every 24 hours for four consecutive days. Primary human GBM cells from patient samples were cultured in the following growth media: DMEM-F12 (Mediatech), 20 ng/ml EGF and bFGF (Gibco), B27 supplement (Life Technologies) and one unit per ml penicillin-streptomycin (Gibco). After 7 to 10 days spheroids were passaged by trypsin treatment and mechanical disruption using a 1 ml syringe and a 23-gauge needle, and dissociated cells were re-plated in fresh growth media. Nrp1-dpendent GSC growth and survival was quantified using the Alamar Blue viability reagent (Promega). HUVECs and complete growth media were purchased from ScienCell, Inc.

Approval for the use of human specimens was obtained from the Institutional Review Board (IRB) at the University of Texas MD Anderson Cancer Center. The IRB waived the requirement for informed consent for previously collected residual tissues from surgical procedures stripped of unique patient identifiers according to the Declaration of Helsinki guidelines. Archived human GBM samples collected before and after bevacizumab treatment were obtained through a protocol approved by the IRB at the University of California San Francisco. TCGA database was queried to compare the expression of Nrp1 between low-grade tumor samples and GBM subtypes. The TCGA database was accessed online through Project Betastasis (http://www.betastasis.com/). The TCGA-based coincidental expression analysis for TGFBR2 and NRP1 in GBM samples was performed using Project Betastasis.

### Experimental mice for tumor studies

We utilized relevant mouse strains to resolve specific and novel in vivo questions related to mechanisms of brain tumor growth and resistance to therapy. All animal procedures were conducted under peer-reviewed Institutional Animal Care and Use Committee (IACUC)-approved protocols. The MD Anderson Cancer Center IACUC approved all animal experiments conducted in this study. The IACUC has adopted guidelines to instruct investigators and others working with laboratory animals on the potential for commonly performed procedures to cause pain/stress. NIH Guidelines, PHS Policy, and USDA Regulations require the assessment of the potential for pain or stress in association with any procedure performed on a laboratory animal. Use of animals in research is minimized if in vitro research applications are possible.

Healthy male NCR-nu/nu mice between 6–12 weeks of age were purchased from Jackson Laboratories. NCR-nu/nu mice were injected intracranially with 2 x 10^5^ LN229 GBM cells expressing GFP as well as Nrp1 shRNAs (n = 10 mice) or non-targeting control shRNAs (n = 10 mice) as described previously [27]. Alternatively, 1 x 10^5^ GSC6-27 cells expressing Nrp1 shRNAs (n = 5 mice) or non-targeting control shRNAs (n = 5 mice) were injected intracranially. Mice were anesthetized by intraperitoneal injection of a mixture of Ketamine/ Xylazine/ Acepromazine (100mg/kg + 2.5mg/kg + 2.5mg/kg body weight) using a 1 ml syringe and sterile 22 G needle. At these dosages the mice remain deeply anesthetized for approximately 30 minutes, which allows sufficient time to perform the intracranial injections. At 5 minutes post-anesthesia, lower limb reflexivity was checked: deeply anesthetized animals will not response to a toe pinch. Corneal reflex was also be used to assess depth of anesthesia during surgery. After surgery, mouse were kept warm with gauze pads and a heat lamp and monitored until recovering fully from the procedure. No animals were exposed to unreasonable pain or distress. Analgesia in the form of subcutaneous injections of Buprenorphine SR was administered as necessary based on routine veterinary care guidelines. Mice were monitored daily for the development of brain tumor-related deficits, including lack of coordination, lethargy and cachexia. Upon the development of tumor-related deficits in the first animal, all mice in the cohort were immediately euthanized by intraperitoneal injection of a lethal dose of Avertin (1 mg/10g body weight). The chest cavity was exposed by an incision above the diaphragm. A 22G needle was inserted into the left ventricle followed by continued perfusion with 4% PFA/PBS. Brains were post-fixed overnight followed by experimental analyses.

For subcutaneous tumor experiments, 5 x 10^5^ U87 GBM cells were implanted into the flank of NCR-nu/nu athymic mice (n = 5) in a 100 μl suspension of growth factor-reduced matrigel (BD Biosciences) and serum free culture media. To develop U87-bev and U87-IgG xenografts, five subcutaneous U87 tumors were treated with bevacizumab (10 mg/kg) or IgG from human serum (Sigma) for four weeks. The largest tumor was extracted, dissociated and then reinjected subcutaneously (n = 5 NCR-nu/nu mice), at which point the process was repeated two more times. The final xenografts were named U87-bev and U87-IgG based on the agent they had been serially treated with. Mice with tumors were monitored daily. Euthanasia occurred when mice reached institutional euthanasia criteria (2.1 cm maximal dimension or tumor symptoms). These animal procedures were approved by the IACUC at MD Anderson Cancer Center. The final tumor lysates collected from bevacizumab-treated and IgG-treated mice represent the bevacizumab-resistant U87-bev^R^ and -sensitive U87-bev^S^ xenografts, respectively.

### Brain tumor volume and blood vessel density quantitation

Brains were dissected, embedded in paraffin, and sectioned coronally. To compare the size of tumors formed by LN229 cells expressing Nrp1-specific shRNA versus NT shRNA, FFPE brain sections were stained with hematoxylin and eosin. The cross-sectional area of each tumor was measured at its largest point. Measurements from at least five tumors were averaged from either condition. To quantify Nrp1-dependent vascular densities in LN229 GBM-bearing mice (n = 3 mice per cell type injected), numbers of CD31-expressing blood vessels were counted in five randomly selected 20x fields. Alternatively, anti-GFAP immunohistochemical staining intensity was quantified in randomly selected fields from mice harboring tumors formed from LN229 cells (n = 3 control tumors and n = 3 Nrp1 shRNA tumors). We quantified the relative Nrp1-dependent sizes of GSC6-27-generated brain tumors (n = 3 mice per cell type injected) by measuring GFP fluorescent signal with ImageJ in brain sections sliced coronally in 1 mm intervals through the injection point (n = 3 slices per tumor).

### Antibodies

Immunoblotting was performed on detergent-soluble lysates using standard protocols. The following primary antibodies were used: rabbit anti-pSMAD3 (pSer423/425, Cell Signaling, 1:1000), rabbit anti-total SMAD2/3 (Cell Signaling, 1:1000), human specific goat anti-Nrp1 (C-19, Santa Cruz, 1:500), goat anti-mCherry (ThermoFisher PA5-34975, 1:1000), rabbit anti-Nrp2 (Santa Cruz sc-5542, 1:1000), rabbit anti-phospho-Histone H3 (Cell Signaling, 1:500), and mouse anti-αν integrin (BD cat#611012, 1:250). Immunoblots were overlayed with donkey anti-rabbit 800, donkey anti-mouse 680, and donkey anti-goat 800 secondary antibodies purchased from Licor (1:10,000). Antibodies were added in blocking buffer comprising 3% bovine serum albumin (BSA) in Tris buffer saline containing 0.1% Tween-20 (TBST). Immunofluorescence and immunohistochemistry was performed on formalin-fixed paraffin-embedded (FFPE) tissue according to standard protocols using the following primary antibodies: rat anti-CD34 (GeneTex cat#GTX28158, 1:200), goat anti-IBA1 (R&D cat#AF2105, 1:50), rabbit anti-GFAP (DAKO cat#Z0334, 1:500), human-specific goat anti-vimentin (R&D cat#AF2105, 1:50), and rabbit anti-TGFβR2 (Santa Cruz, 1:1000). Alexa-conjugated goat anti-rabbit 488, goat anti-rabbit 594, and goat anti-mouse 594 secondary antibodies (1:200) were used. Antibodies were incubated in blocking buffer comprising 1% bovine serum albumin (BSA) in phosphate buffer saline containing 0.1% TritonX-100 (PBST).

Nrp1 expression was analyzed in FFPE human tissue samples by incubating slides with in PBS containing 20 μg/ml Proteinase K for 20 minutes at room temperature. Primary antibody overlay was performed with rabbit anti-Nrp1 (Santa Cruz sc-5541, 1:50), which reacts with Nrp1 from multiple species, in blocking buffer comprising 10% swine serum in PBST. Slides were incubated in PBS containing 3% hydrogen peroxide, and then incubated with swine anti-rabbit biotinylated (DAKO cat# E0353, 1:250) secondary antibody in blocking buffer. Tissue staining was visualized using the Vector Labs Vectastain ABC (cat#PK-4000) and Vector Labs DAB (cat#SK-4100) reagent kits, and counterstained with hematoxylin. Images of stained tissue sections and cells were captured using an Olympus confocal microscope.

### Plasmids and lentiviruses

pGIPZ lentiviral vectors encoding human Nrp1-specific or non-targeting (NT) shRNAs (Dharmacon) have been described elsewhere [25]. Plasmids were co-transfected into HEK293T with vectors encoding gag, pol, and VSV/G env genes. Virus particles were harvested and used to transduce Nrp1-specific or NT-shRNA into LN229. RNAi-mediated silencing of Nrp1 was validated by immunoblotting. The Neuropilin1-Cherry plasmid was purchased from Addgene, Inc. The Cherry control plasmid was generated by excising Nrp1 cDNA with AgeI restriction enzyme and re-ligating the vector. LN229 cells were transfected with Nrp1-Cherry or Cherry control vectors. Stably transfected cells were selected with 1 mg/mL G418 (Gibco cat#11811–023). The plasmid containing the full-length rat Nrp1 cDNA was provided by Dr. Chenghua Gu (Harvard University), and was mutated to generate the Nrp1 construct lacking the cytoplasmic tail. The plasmid with the human Nrp1 cDNA for HEK-293T cell transfections were kindly provided by Dr. Lee Ellis (MD Anderson Caner Center). The plasmid expressing myc-tagged TGFβR2 was provided by Dr. Elaine Fuchs (Rockefeller University). More details about the Nrp1 and Tgfbr2 plasmids are described elsewhere [25]. The NRP1 primer sequences used for the qRT-PCR experiments are 5' TGT GCC AAA GAT GTC AGA GA 3' and 5' ACC TGG TGT TTT CTG TCC AC 3'.

### TGFβ and VEGF-A signaling assays

LN229 cells were seeded onto 60mm plates and serum starved overnight. Cells were treated with 0.1 ng/ml rhTGFβ1 (R&D cat#240-B-002/CF) for 0, 15, and 30 minutes. For VEGF-A competition experiments, 100 ng/ml rhVEGFA (Biolegend cat#583704) was used. Cells were lysed in 50 mM Tris, pH 7.4, 150 mM NaCl, 1% NP40, 1 mM EDTA containing a cocktail of protease and phosphatase inhibitors (Roche), and analyzed by western blotting. Band densities were quantified using ImageJ software.

### TGFβR2 internalization experiments

LN229 GBM cells expressing GFP as well as control shRNAs or Nrp1 shRNAs were plated on laminin-coated slides (3 x 10^3^ cells per 8-well chamber slide). Cells were serum starved overnight, treated with 5 ng/ml TGFβ1 for 0, 5, 15, and 30 minutes. After stimulation cells were immediately fixed with 4% PFA/PBS in the absence of detergent to prevent membrane permeabilization. Anti-TGFβR2 primary antibodies and goat anti-rabbit Alexa594-conjugated secondary antibodies were use to label TGFβR2 on the cell surface. Three independent experiments were performed and 3 wells were stimulated with TGFβ1 for each time point. Anti-TGFβR2 fluorescence signal was quantified at each time point for at least 10 randomly selected cells (based on DAPI staining) per well. 80x magnification images were taken for analysis. ImageJ software was used to calculate the average TGFβR2 staining intensity per cell.

### Statistical analysis

All data represented herein were performed in replicates of three or more and are presented as the mean ± standard deviation, unless otherwise indicated. Differences among groups were analyzed using one-way analysis of variance. When overall analysis revealed significance among groups, means were compared and tested using Tukey’s post-hoc analysis. Statistical significance was set at P < 0.05. All statistical analyses were performed in SigmaPlot 12.0 software (Systat Software, Inc., San Jose, CA).

## Results

**Fig 1:**
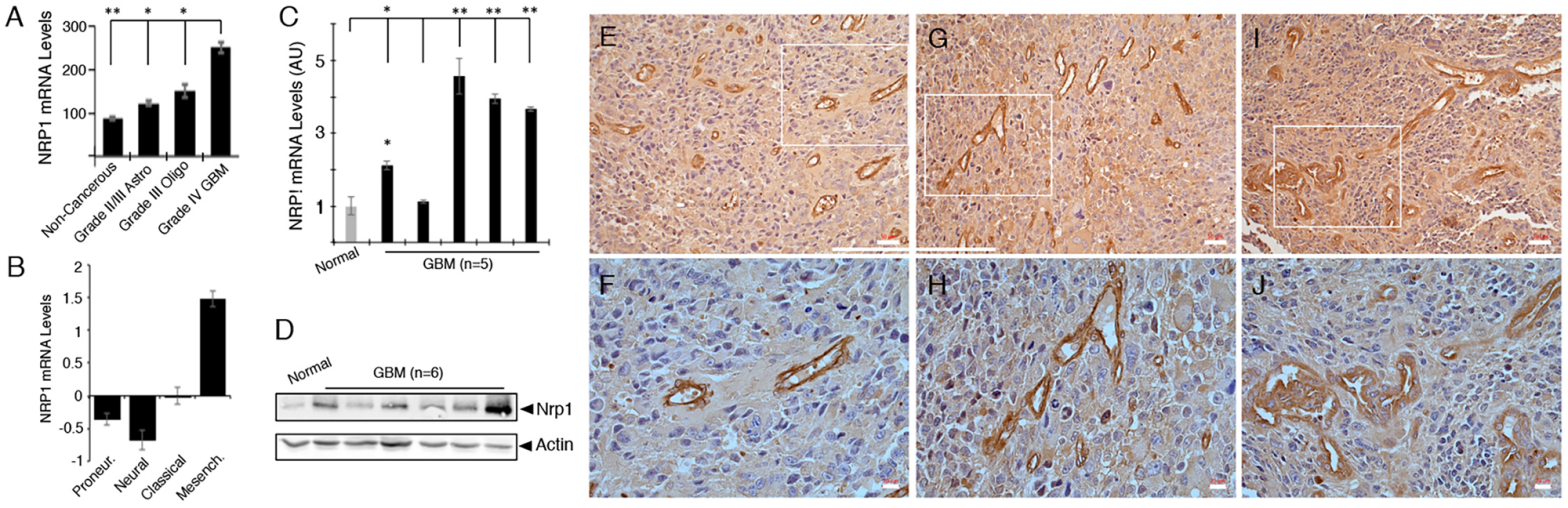
NRP1 RNA and protein expression are upregulated in human GBM tissues. **(A);** Analysis of GBM TCGA reveal that NRP1 RNA expression levels show progressive upregulation depending on tumor grade, with GBM samples expressing the highest levels of NRP1, *p<0.05 and **p< 0.01 for GBM in relation to lower-grade tumors and non-cancerous control brain. **(B);** NRP1 is a molecular marker for the mesenchymal GBM sub-type based on analysis of the GBM TCGA database. **(C);** Quantitative RT-PCR using RNA from a non-cancerous control brain tissue (normal) or five different GBM samples, *p<0.05 and **p< 0.01 for GBM in relation to normal control brain tissue. **(D);** Anti-Nrp1 immunoblot using detergent-soluble lysates from non-cancerous control brain tissue or six different freshly resected GBM specimens. Note that in comparison to control brain, Nrp1 protein levels are higher in five of the six tumor samples. **(E-J);** Immunohistochemistry stains using an anti-Nrp1 antibody reveal robust Nrp1 protein expression in three different human GBM samples. Note that Nrp1 protein is expressed in GBM cells as well as in intratumoral blood vessels. Lower panels (F, H, J) are higher magnification images of boxed areas in upper panels (E, G, I). Scale bars, 50 μm in D-F and 20 μm in G-H. All error bars represent standard deviation.

To determine functions for Nrp1 in GBM we first analyzed levels of Nrp1 gene expression in human brain tumor samples by querying The Cancer Genome Atlas (TCGA) GBM database. As shown in Fig 1A, NRP1 RNA levels are elevated approximately two-fold in human GBM samples versus normal human brain. NRP1 RNA levels are also significantly higher in GBM compared to grade II/III astrocytomas or grade III oligodendrogliomas. TCGA analysis reveals that NRP1 is a molecular marker for the mesenchymal GBM sub-type (Fig 1B). Quantitative real time PCR also confirmed elevated NRP1 RNA expression in four of five GBM samples analyzed, in comparison to a non-cancerous brain tissue control (Fig 1C). Mesenchymal tumors are distinguished from other molecular subtypes by enhanced expression of genes involved in pathological angiogenesis, hypoxia, and necrosis, including VEGF-A and ECM components such as fibronectins and angiopoietins [28–30]. Querying the open access IVY GBM Atlas Project (GAP) revealed that Nrp1 RNA is expressed in different tumor regions including the tumor vasculature. In contrast, analysis of the closely related Neuropilin 2 (NRP2) gene reveals no upregulation in GBM samples versus normal brain or lower grade brain tumors (S1 Fig). Like NRP1, NRP2 RNA expression correlates with the mesenchymal GBM sub-type and coincides, in part, with NRP1 expression in different microdissected GBM regions (S1 Fig). Segregation of primary GBM patients based on differential expression of NRP1 (high versus low quartiles) did not reveal differences in overall survival (S1 Fig).

Next, we used an anti-Nrp1 antibody to immunoblot detergent-soluble lysates from a non-cancerous brain tissue sample or from six different primary GBM samples. Nrp1 protein levels were elevated in five of the six tumor lysates analyzed (Fig 1D). We also used an anti-Nrp1 antibody to immunolabel formalin fixed paraffin embedded tissue sections from surgically resected human GBM samples. All fixed surgical specimens were from patients with primary GBM that had not received prior radiation or chemotherapy treatments. As shown in Fig 1E–1J and S2 Fig, Nrp1 protein was expressed in tumor cells as well as in vascular endothelial cells comprising intratumoral blood vessels. Nrp1 protein was also detected by immunohistochemistry in blood vessels of the developing human brain (S3 Fig).

**Fig 2:**
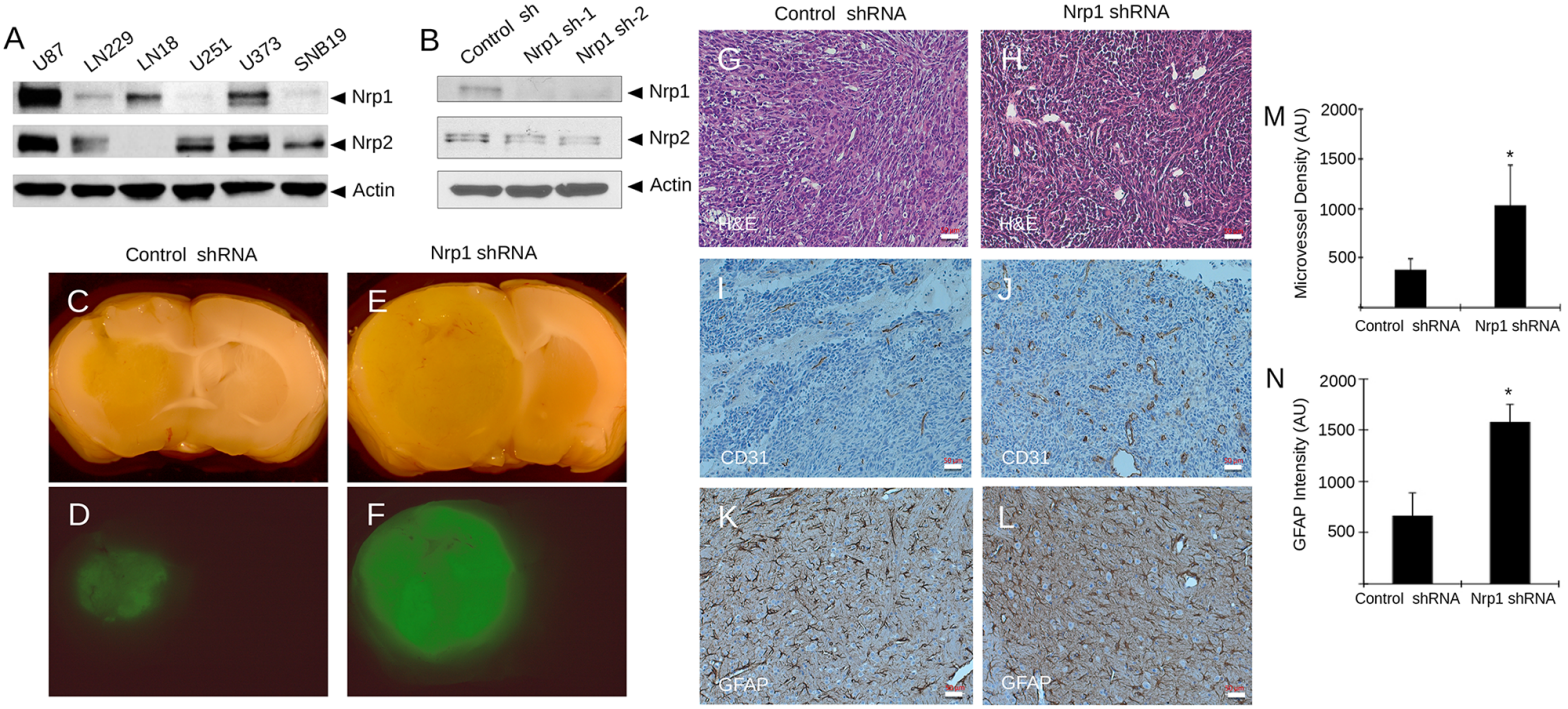
Genetically targeting NRP1 leads to enhanced GBM growth and progression in vivo. **(A);** Immunoblot analyses of detergent-soluble lysates from six different human GBM cell lines reveals varying levels of Nrp1 and Nrp2 protein expression. **(B);** RNAi-mediated targeting of NRP1 in LN229 GBM cells with two different lentiviral-expressed shRNAs leads to reduced expression of Nrp1 protein, but not Nrp2 protein, as revealed by immunoblotting. **(C-F);** LN229 cells stably expressing control (non-targeting) shRNAs (C, D) or shRNAs targeting Nrp1 (E, F) were injected intracranially (n = 5 mice per cell type) and brain tumor xenografts were subsequently analyzed by bright field (C, E) and GFP fluorescence imaging (D, F). Note the obvious Nrp1-dependent differences in tumor size in the representative images. **(G-L);** Analysis of Nrp1-dependent angiogenesis and reactive gliosis in brain tumor xenografts by H&E staining (G, H), anti-CD31 (I, J) and anti-GFAP (K, L), respectively. Scale bars, 20 μm. **(M, N);** Quantitation of blood vessel densities based on CD31 expression (M) and tumor-associated astrocyte based on GFAP expression (N) in brain tumors generated from LN229 cells expressing control shRNAs or Nrp1 shRNAs, *p<0.05 for Nrp1 shRNA tumors in comparison to control shRNA tumors. All error bars represent standard deviation.

We also compared Nrp1 and Nrp2 protein levels in six different human GBM cell lines. As shown in Fig 2A, we detected varying levels of Nrp1 and Nrp2 proteins, with some cell lines such as U87 and LN229 expressing relatively high levels of both proteins, and other cell lines such as LN18 and U251/SNB19 expressing one Nrp protein but not the other. We used lentiviruses expressing GFP and shRNAs targeting different regions of NRP1 (n = 2 different shRNAs) to silence gene expression in LN229 GBM cell pools. This strategy resulted in a near complete absence of Nrp1 protein expression without impacting Nrp2 expression (Fig 2B). Analysis of LN229 cell proliferation in vitro did not reveal a significant difference in the control shRNA cells versus Nrp1 shRNA cells (S4 Fig). Next, we intracranially implanted LN229 cells to analyze Nrp1-dependent brain tumor growth and progression. In comparison to control cells (n = 10 NCR-nu/nu mice), LN229 cells expressing Nrp1 shRNAs (n = 10 NCR-nu/nu mice) generated significantly larger intracranial tumors (Fig 2C–2F and S4 Fig), suggesting a critical role for Nrp1-dependent signaling mechanisms in GBM growth and progression. Quantitation of GBM volumes by measuring cross-sectional areas in serial sections revealed that tumors derived from LN229 cells expressing Nrp1 shRNAs were nearly two-fold larger than tumors formed from control LN229 cells (S4 Fig). Analysis of tumor cell proliferation in situ by double immunofluorescence using a human-specific vimentin antibody and anti-pSer10 Histone H3, a marker for mitotic cells, did not reveal Nrp1-dependent differences proliferation. Immunohistochemical analysis revealed that LN229 tumors expressing control shRNAs were vascularized (Fig 2G–2J); however, tumors lacking Nrp1 expression contained blood vessels with more abundant and displayed dilated morphologies that were often hemorrhagic (Fig 2H and 2J and S4 Fig). LN229 xenograft tumors expressing control and Nrp1 shRNAs both contained reactive astrocytes (Fig 2K and 2L and S5 Fig). Quantitation of blood vessel density based on CD31 expression and astrocytes based on GFAP expression revealed that tumors derived from Nrp1 shRNA cells were more vascularized and contained higher numbers of reactive astrocytes (Fig 2M and 2N). Nrp1-dependent differences in intratumoral microglial cells were not detected (S5 Fig). In addition, control and Nrp1 shRNA tumors did not display obvious difference in invasion into the surrounding microenvironment (S5 Fig).

In addition to analyzing Nrp1 regulation of tumor growth in the LN229 GBM cell line, we also analyzed primary stem-like GBM cells (GSCs) derived from patient tumors. These cells growth as neurosphere-like spheroids in serum-free media and generate highly malignant tumors in the mouse brain [31]. We detected variable levels of Nrp1 protein expression in six different GSC preparations (S6 Fig). We selected GSC7-2 spheroids, which express relatively high levels of Nrp1 protein, for gene silencing using lentiviruses expressing GFP and shRNAs (S6 Fig). We did not detect quantifiable differences in Nrp1-dependent GSC proliferation and spheroid formation (S6 Fig). Intracranial injections of GSC7-2 cells expressing non-targeting control shRNAs generated large brain tumors (S6 Fig). In contrast, implantation of GSCs expressing Nrp1 shRNAs led to larger, more diffuse intracranial tumors (S6 Fig). Hence, Nrp1 has growth-suppressive functions in both the LN229 GBM cell line and in primary GSCs.

**Fig 3:**
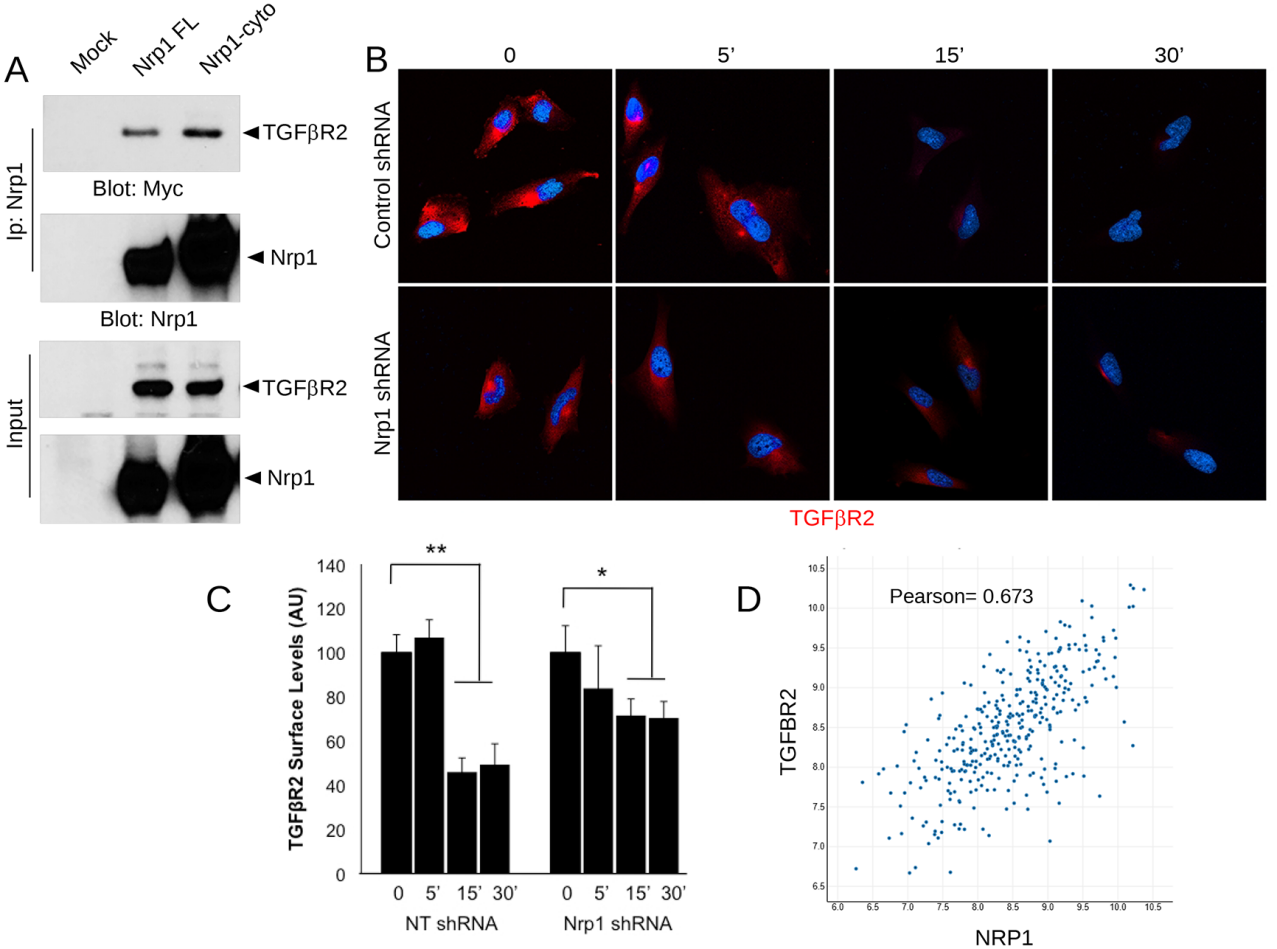
Nrp1 interacts with TGFβR2 to promote receptor internalization in GBM cells. **(A);** HEK 293T cells were transfected with myc-tagged TGFβR2 and full-length Nrp1 or Nrp1 lacking the cytoplasmic domain. Protein-protein interactions were then analyzed in detergent-soluble lysates by co-immunoprecipitation. Note that TGFβR2-myc interacts with Nrp1 independently of the Nrp1 cytoplasmic tail. **(B);** LN229 GBM cells expressing control shRNAs (top) or Nrp1 shRNAs (bottom) were treated with TGFβ1 for varying times (indicated along top of panel). Cell surface levels of TGFβR2 protein were analyzed by immunofluorescently labeling fixed, non-permeabilized cells with anti-TGFβR2 antibodies. **(C);** Quantitation of Nrp1-dependent TGFβR2 protein internalization in LN229 cells following TGFβ1 treatment. Error bars represent standard deviation, *p<0.05 for control shRNA samples (time 0) versus 15 and 30 minutes samples, **p<0.01 for Nrp1 shRNA samples (time 0) versus 15 and 30 minute treatments. **(D);** Two gene scatter plot showing coincident NRP1 and TGFBR2 RNA expression in TCGA GBM samples. The results are statistically significant based on a Pearson’s coefficient of 0.673. All error bars represent standard deviation.

Prior reports have shown that Nrp1 can serve as a TGFβ co-receptor and modulate TGFβ receptor signaling in various cell types [32]. Transfection and co-immunoprecipitation experiments revealed the formation of protein-protein complexes between Nrp1 and TGFβR2 (Fig 3A). The interaction between Nrp1 and TGFβR2 was independent of the Nrp1 cytoplasmic domain, since truncation of this region did not impact interactions with TGFβR2, suggesting that the Nrp1 transmembrane and/or extracellular region mediate binding to TGFβR2 protein. TGFβ interactions with the TGFβR1/TGFβR2 complex leads to receptor internalization and continued intracellular signaling via Smads [32]. Indeed, in endothelial cells and tumor cells Nrp1 can promote the internalization of the VEGFR2 cell surface protein [33]. Therefore, we next analyzed Nrp1-dependent internalization of TGFβR2, the common TGFβ receptor that dimerizes with multiple type 1 receptors [34]. As shown in Fig 3B, when control LN229 cells were treated with TGFβ1 we detected a time-dependent internalization of TGFβR2 protein, as revealed by immunofluorescently labeling fixed LN229 cells with an anti-TGFβR2 antibody. Cells not permeabilized with detergent, to allow for labeling TGFβR2 on the cell surface, but not intracellular pools of protein. Reduced time-dependent TGFβR2 internalization was detected in LN229 cells expressing Nrp1 shRNAs (Fig 3C and 3D). In contrast, overexpression of a Cherry-tagged Nrp1 protein in LN229 enhanced the rate of TGFβR2 internalization in response to TGFβ1 (S8 Fig). While the C-terminal Cherry tag likely blocks interactions with the cytoplasmic protein Gipc1 [35] and VEGF receptor signaling, other reports have shown that truncation of the Nrp1 cytoplasmic tail does not impact interactions between Nrp1 and TGFβ receptors or impact canonical TGFβ signaling [24, 25].

Prior reports have shown that Nrp1 can modulate canonical TGFβ receptor signaling in various cell types [32]. To determine how Nrp1 may impact TGFβ signaling we analyzed Nrp1-dependent Smad3 phosphorylation in LN229 cells. As shown in S7 Fig, silencing Nrp1 in GBM cells leads to a reduction in TGFβ receptor signaling, as determined by quantifying levels of phosphorylated Smad3 (pSmad3) protein following treatment with TGFβ1. This was most apparent at the earliest time points analyzed (0, 5 and 15 minutes), although the Nrp1-dependent kinetics of Smad3 phosphorylation were similar with time. Forced expression of Cherry-tagged Nrp1 led to a significant increases in pSmad3 levels in response to TGFβ1 treatment (S8 Fig). In addition, forced expression of Nrp1 in HEK-293T cells led to a time-dependent increase in pSmad2 and pSmad3 levels (S7 Fig). Interestingly, the lentiviral-expressed Nrp1 shRNAs that caused diminished Smad3 phosphorylation in GBM cells did not generate a similar response in human umbilical vein endothelial cells (HUVECs). HUVECs were analyzed because they express high levels of endogenous Nrp1 protein and display robust Smad phosphorylation [24, 25]. Phosphorylated Smad3 levels were actually higher in HUVECs expressing Nrp1 shRNAs versus control shRNAs (S9 Fig). In addition, we did not detect Nrp1-dependent changes in TGFβR2 internalization in HUVECs (S9 Fig). HUVECs expressing Nrp1 shRNAs also displayed defects in F-actin cytoskeletal organization (S9 Fig). Hence, the Nrp1-dependent effects on TGFβ receptor internalization and canonical signaling are highly dependent on cell context.

**Fig 4:**
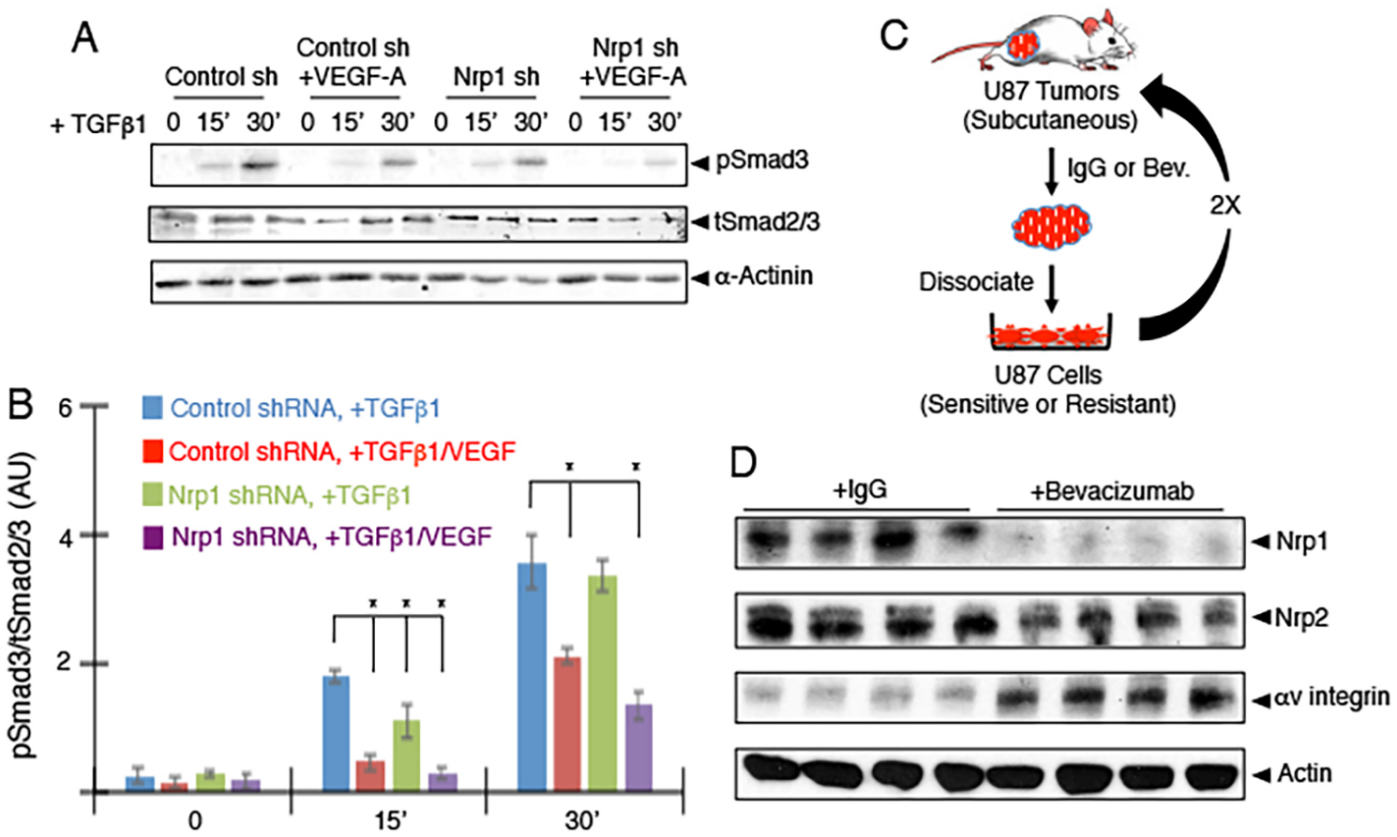
VEGF-A competitively inhibits Nrp1-mediated canonical TGFβ receptor signaling in GBM cells. **(A);** LN229 cells were treated with 1 ng/ml TGFβ1 for varying times in the presence or absence of 100 ng/ml VEGF-A. Detergent-soluble lysates were immunoblotted with anti-pSmad3 antibodies to access Nrp1-dependent canonical TGFβ signaling. Note that VEGF-A competitively inhibits TGFβ-mediated Smad3 phosphorylation in control cells, and these effects are enhanced in LN229 cells expressing Nrp1 shRNAs. **(B);** Quantitation of Nrp1-dependent Smad3 phosphorylation levels in response to TGFβ1 and VEGF-A treatment of LN229 cells. Error bars represent standard deviation, *p<0.05 for control shRNA/+TGFβ1 versus other treatments at 15 minutes and *p<0.05 for control shRNA/+TGFβ1 versus Nrp1 shRNA/+TGFβ1 and Nrp1 shRNA/+TGFβ1/VEGF-A treatments at 30 minutes. All error bars represent standard deviation. **(C);** Diagram showing the experimental strategy to generate drug-resistant U87 GBM cells by selecting for tumor growth in mice followed by bevacizumab treatment. **(D);** bevacizumab-sensitive (U87-bev^S^, n = 4) and bevacizumab-resistant (U87-bev^R^, n = 4) tumor lysates were immunoblotted with human-specific antibodies directed against Nrp1, Nrp2, or αv integrin proteins. Note that Nrp1 protein levels are diminished in U87-bev^R^ tumors, whereas αv integrin levels are increased and Nrp2 levels are unchanged.

Elevated VEGF-A levels and receptor signaling correlate with uncontrolled angiogenesis and hemorrhage in GBM [36]. Activation of TGFβ receptor signaling pathways are reported to inhibit the pro-angiogenic and vascular permeability actions of VEGF-A [37]. Indeed, inhibition of TGFβ signaling or activation of VEGF-A signaling can lead to similar angiogenesis pathologies in the developing brain [38]. Therefore, we next analyzed links between Nrp1-dependent canonical TGFβ receptor signaling via VEGF-A and TGFβs in GBM cells. LN229 were stimulated with TGFβ1 alone or VEGF-A/TGFβ1 in combination and levels of pSmad3 were quantified by immunoblot. As shown in Fig 4A, VEGF-A treatment of control LN229 cells competitively inhibited TGFβ1-induced Smad3 phosphorylation. Silencing Nrp1 with lentiviral-expressed shRNAs showed that VEGF-A effects on TGFβ signaling were Nrp1-dependent (Fig 4B).

Given that Nrp1 can modulate VEGF-A and TGFβ signaling in GBM cells (Fig 4) and that Nrp1 suppresses GBM growth within the context of the brain microenvironment (Fig 2), we next analyzed links between Nrp1 expression and GBM recurrence following neutralization of VEGF-A with bevacizumab. First, Nrp1 protein levels were analyzed in GBM xenograft tumors that were selected *in vivo* for acquired bevacizumab resistance [5]. In brief, xenograft U87 tumors grown subcutaneously were treated twice per week with control IgG or bevacizumab (Fig 4C). Tumors were surgically removed, dissociated, and cells were re-implanted into recipient mice followed by two additional rounds of IgG or bevacizumab treatment to select for bevacizumab-responsive IgG-treated (U87-bev^S^) versus bevacizumab-resistant (U87-bev^R^) GBM cells. In comparison to U87-bev^S^ tumors, human Nrp1 protein was significantly down regulated in all four U87-bev^R^ tumor xenografts, as revealed by immunoblotting lysates with a human-specific anti-Nrp1 antibody (Fig 4D). Nrp2 protein levels remained unchanged in U87-bev^S^ versus U87-bev^R^ tumor lysates. In contrast to Nrp1 down regulation, we detected a significant increase in human αv integrin protein expression in U87-bev^R^ GBM cells, consistent with a previous report [39].

**Fig 5:**
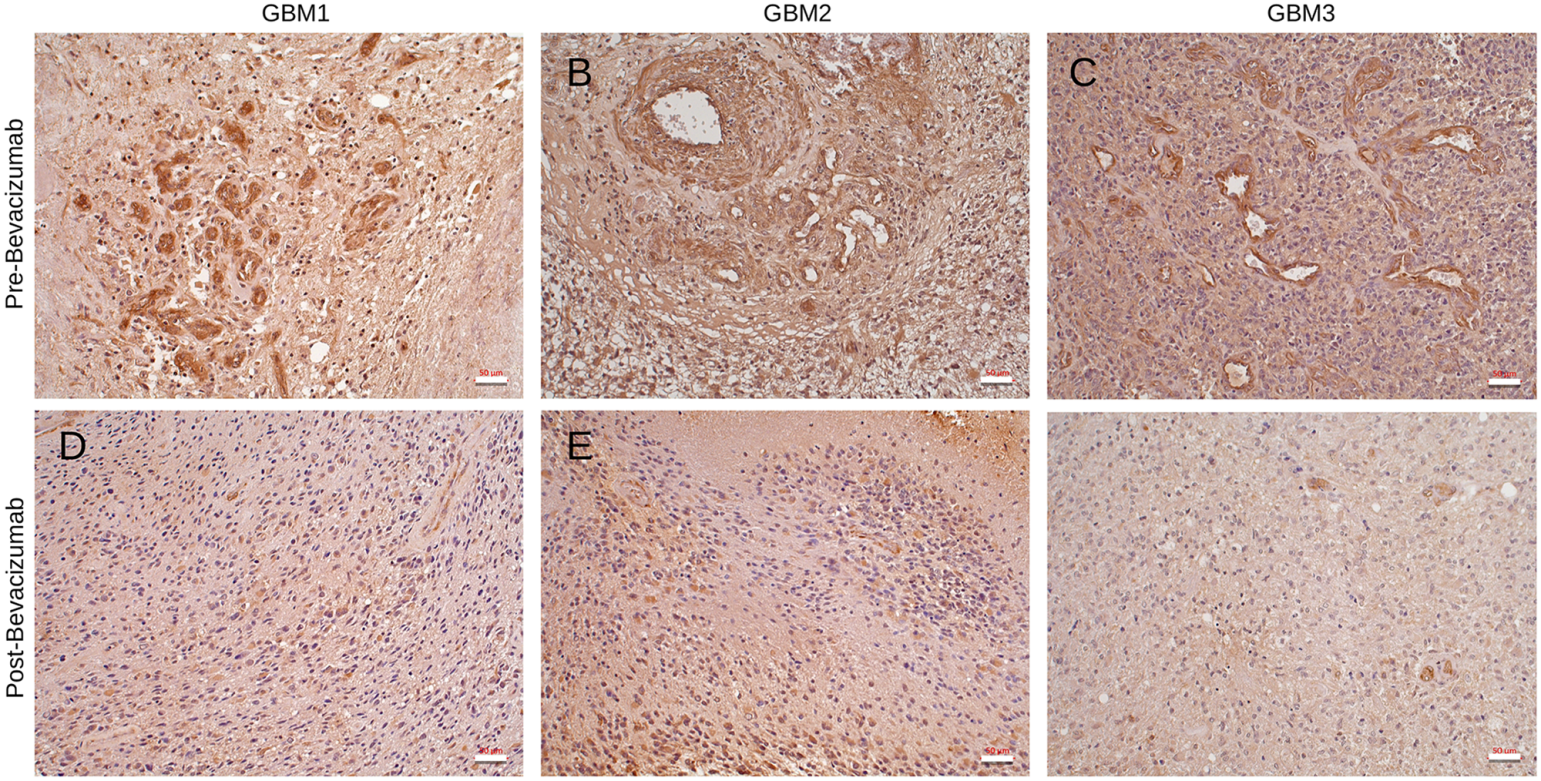
Nrp1 protein expression is down regulated in recurrent GBM following bevacizumab treatment. **(A-F);** Immunohistochemistry was performed with anti-Nrp1 antibodies using matched human GBM surgical resections collected prior to bevacizumab treatment (A-C) and after bevacizumab treatment (D-F). Note that Nrp1 protein levels are diminished in GBM cells and in intratumoral blood vessels in recurrent tumors following bevacizumab therapy.

The subcutaneous tumor models treated with bevacizumab do not rule out roles for mouse VEGF-A in the microenvironment. Therefore, we next studied spatiotemporal patterns of Nrp1 protein expression in fixed GBM samples by immunohistochemistry. Matched brain tumor samples were analyzed from three different patients undergoing surgical resection prior to bevacizumab treatment as well as post-bevacizumab treatment (recurrent tumors). As shown in Fig 5A–5C, in GBM samples collected prior to bevacizumab treatment (bevacizumab-responsive) Nrp1 protein was robustly expressed in tumor cells as well as in intratumoral blood vessels. In contrast, in all three recurrent GBM samples (bevacizumab-resistant) we detected a striking reduction in Nrp1 protein expression in tumor cells as well as in many intratumoral blood vessels (Fig 5D–5F).

**Fig 6:**
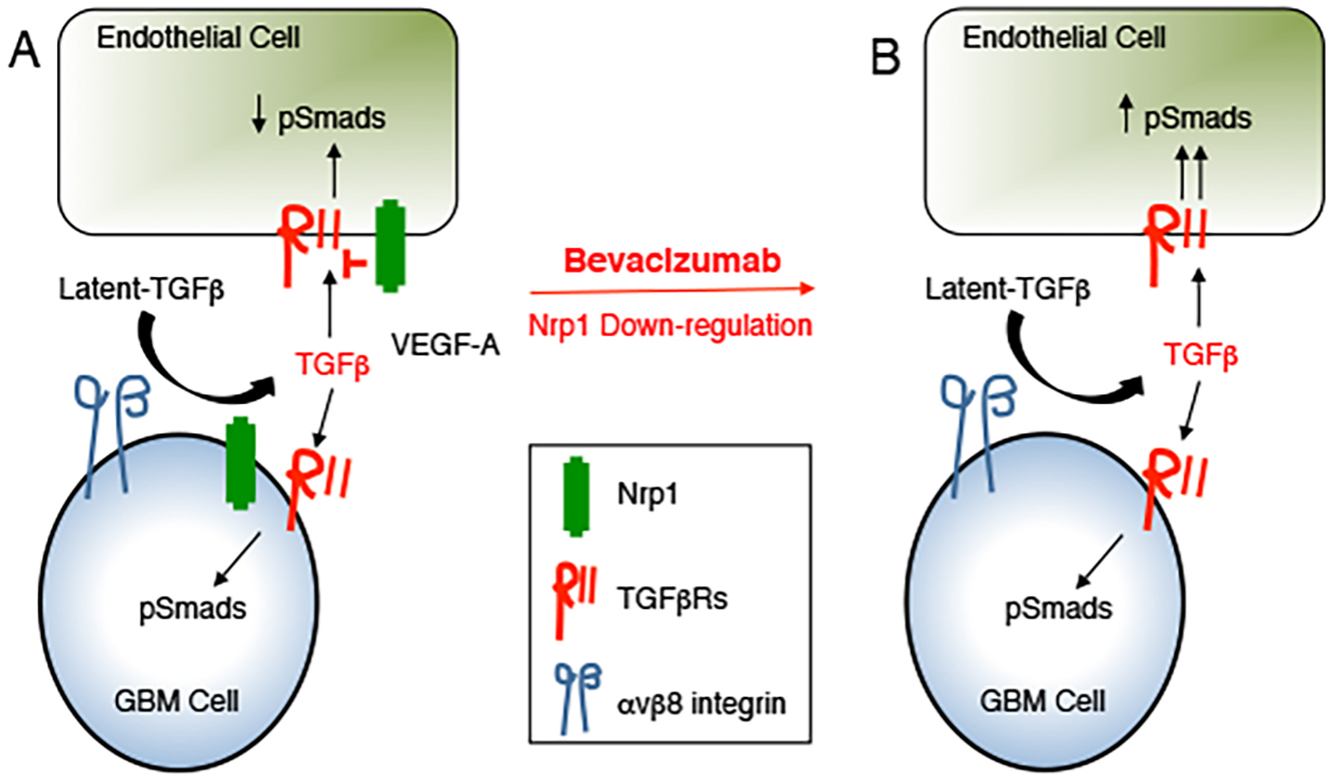
A model for Nrp1-dependent regulation of TGFβ signaling in GBM. **(A);** Nrp1 is expressed in GBM cells, where it along with canonical TGFβ receptors, serves as a co-receptor for TGFβs. Nrp1 enhances canonical TGFβ receptor signaling in GBM cells. In addition, Nrp1 in GBM cells can influence TGFβ receptor signaling in vascular endothelial cells and probably other cells in the tumor microenvironment. Nrp1 can also regulate VEGF-A signaling in GBM cells and in endothelial cells of the tumor microenvironment. **(B);** Targeting the VEGF-A pathway with bevacizumab disrupts the Nrp1-dependent balance in VEGF-A versus TGFβ signaling pathway by down-regulating Nrp1 expression In GBM cells, thus promoting neovascularization and tumor recurrence in response to VEGF-A neutralization.

Collectively, these data reveal that tumor growth, progression and recurrence following anti-vascular therapies such as bevacizumab are dependent, in part, on the Nrp1-TGFβ signaling pathway. We propose a model in which Nrp1 promotes interactions between GBM cells and blood vessels in the brain tumor microenvironment via the TGFβ pathway as well as VEGF-A. This leads to differential Nrp1-dependent TGFβ receptor signaling in tumor cells versus endothelial cells and possibly other stromal cells. Diminished expression of Nrp1 in GBM cells leads to deregulation of this signaling balance, leading to VEGF-A independent angiogenesis and tumor recurrence following bevacizumab treatment (Fig 6).

## Discussion

Here, we have analyzed functions for Nrp1 in GBM growth, progression and recurrence after anti-angiogenic therapy. Our data reveal the following novel findings: (i) NRP1 RNA and protein expression increases as brain tumors progress from low grade to high grade GBM (Fig 1); (ii) RNAi-mediated silencing of NRP1 in human GBM cells leads to enhanced tumor growth in vivo that is due, in part, to increased angiogenesis and the development of intratumoral vascular pathologies (Fig 2); (iii) Nrp1 in GBM cells stimulates TGFβ receptor internalization and signaling (Fig 3); (iv) Nrp1 competitively modulates TGFβ and VEGF-A signaling pathways in GBM cells (Fig 4); and (v) Nrp1 is down-regulated in recurrent GBM in response to acquired resistance to the anti-VEGF-A therapeutic antibody bevacizumab (Figs 4 and 5 and model in Fig 6).

Nrp1 as a TGFβ receptor signaling component has been reported previously in fibroblasts, endothelial cells and immune cells, and has been implicated as a driver of tumor progression [20, 40]. Despite these findings, little is known about the mechanisms underlying Nrp1-mediated TGFβ signaling. We sought to elucidate the roles for this pathway in human LN229 GBM cells that express Nrp1 and show TGFβ-responsive signaling. RNAi-mediated NRP1 silencing unexpectedly leads to enhanced tumor growth, suggesting that Nrp1 suppresses proliferation in GBM cells in vivo. We initially expected that the enhanced growth of tumors lacking NRP1 would be due to GBM cell-intrinsic pathways related to decreased TGFβ signaling. Indeed, activation of the TGFβ signaling pathway has previously been shown to suppress proliferation of GBM cells [9]. However, we do not detect Nrp1-dependent GBM cell growth defects in vitro or in vivo. Reduced Nrp1 expression in LN229 GBM xenografts, however, leads to increased intratumoral angiogenesis and vascular permeability, suggesting additional functions for tumor cell-expressed Nrp1 in cross-talk with stromal components in the GBM microenvironment. The extracellular region of Nrp1 likely facilitates heterophillic interactions between tumor cells and endothelial cells in angiogenic blood vessels. Hence, in GBM cells that lack Nrp1 via RNAi-mediated silencing, the increased intratumoral blood vessel densities are probably the result of aberrant paracrine/trans Nrp1-mediated regulation of TGFβ versus VEGF-A signaling in vascular endothelial cells. Therefore, we propose that the increased sizes of orthotopic brain tumors that lack Nrp1 are, in part, the outcome of increased VEGF-A-driven angiogenesis, enabling more rapid GBM progression. It will be interesting to test this possibility by treating mice harboring Nrp1-deficient brain tumors with inhibitors of the VEGF-A pathway.

Nrp1-dependent angiogenesis pathologies in GBM are more likely related to latent-TGFβ activation and signaling. In GBM cells Nrp1 promotes TGFβ signaling, whereas in endothelial cells Nrp1 suppresses TGFβ signaling. Hence, the exact cellular context of the Nrp1 interaction with TGFβ receptors plays a critical role in determining TGFβ signaling outputs. A prior report has shown that cis interactions between Nrp1 and VEGF receptors can enhance signaling, whereas trans interactions with Nrp1 can inhibit VEGF receptor signaling by promoting internalization [33]. Interestingly, our data reveal that Nrp1 modulates TGFβR2 internalization and canonical TGFβ signaling in GBM cells, but not in endothelial cells. While we do not know the exact mechanisms underlying Nrp1-mediated TGFβR2 internalization or recycling, it is likely that the intracellular scaffolding protein Gipc1, which binds directly to a PDZ domain in the Nrp1 cytoplasmic tail [35], is involved in these processes.

Nrp1-mediated TGFβ signaling in GBM cells is competitively blocked by treatment with VEGF-A. These results match other reports showing that VEGF-A and TGFβ ligands compete for binding to Nrp1 [22]. Autocrine VEGF-A signaling is reported to increase proliferation and suppress invasion in GBM cells [41]; therefore, elevated Nrp1 expression in GBM cells may be one mechanism to overcome competition between VEGF-A and TGFβ ligands, thus enabling the activation of both pathways simultaneously to promote tumor progression. A recent report has shown that silencing Nrp1 diminished TGFβ signaling in pancreatic adenocarcinoma cells, but also inhibited TGFβ1-induced Smad2 phosphorylation in endothelial cells (HUVECs) and blocked endothelial to mesenchymal transition and fibrosis [42]. This is in contrast to our data (S9 Fig) and a prior report showing that Nrp1 silencing leads to enhanced TGFβ-induced Smad phosphorylation [24]. These differential responses in HUVECs may be due to patient-specific differences in primary endothelial cell cultures, or could be related to variability in cell passage number. For example, we have found that highly passaged HUVECs (>5 passages) show altered responsiveness to TGFβ stimulation (data now shown).

Patients treated with the anti-VEGF-A inhibitory antibody bevacizumab or other anti-angiogenic drugs that target the VEGF-A pathway show improvements in progression-free survival. However, overall patient survival rates have not improved due to acquired resistance and tumor recurrence. A significant percentage of patients with recurrent GBM develop unusually robust patterns of invasive cell growth [7, 43]. Molecular studies have shown that bevacizumab alters interactions between VEGFR-2 and c-Met in GBM cells, thus promoting growth and invasive signaling cascades [8]. Recent studies have noted increased expression of TGFβ1 in response to bevacizumab treatment [41]. Using pre-clinical mouse models and matched human specimens, we have found that Nrp1 expression is diminished in bevacizumab-resistant GBM. While these data remain correlative, for example, we have not forcibly overexpressed Nrp1 in bevacizumab-resistant cells and confirmed growth suppression, they suggest that down-regulation of Nrp1 may be one pathway by which tumors promote angiogenesis in the absence of VEGF-A. Targeted therapies against Nrp1 may be useful against bevacizumab-sensitive GBM by inhibiting both VEGF-A and TGFβ signaling. A prior report has shown that overexpression of Nrp1 in U87MG GBM cells stimulates tumorigenesis via activation of the HGF/c-Met signaling pathway [16]. Unlike LN229 GBM cells, U87MG cells generate non-invasive tumors in the mouse brain. U87MG and LN229 cells contain distinct gene mutations [44] and express different biomarkers, including the latent-TGFβ activating integrin αvβ8, which is robustly expressed in LN229 GBM cells but expressed at much lower levels in U87MG cells [45, 46]. It is also possible that in addition to the VEGF-A and TGFβ pathways, Nrp1 is also impacts HGF/c-Met signaling in GBM cells and/or stromal cells.

Lastly, these data showing important roles for Nrp1 signaling in GBM cells and effects on blood vessels in the tumor microenvironment have direct links to what has been reported in brain vascular development. Our group and others have demonstrated that αvβ8 integrin and its latent TGFβ ECM protein ligands are essential for angiogenesis in the developing brain [47–51]. TGFβ1 and TGFβ3 are highly expressed in the developing brain and their combined genetic deletion leads to brain-specific vascular defects that phenocopy those pathologies in αvβ8 integrin mutant mice [49]. Ablation of TGFβ receptor type 2 (TGFβR2) or the type 1 receptor Alk5 in endothelial cells, but not glial cells, results in brain vascular pathologies that are identical to those that develop in αvβ8 integrin and TGFβ1/3 mutant mice [52]. We have shown that Nrp1 balances integrin-activated TGFβ signaling by interacting directly with αvβ8 integrin to block adhesion to latent TGFβs [25]. αvβ8 integrin is highly expressed in primary GBM cells and in many GBM cell lines [46], suggesting functional links between this integrin and Nrp1 in GBM growth and angiogenesis. Selective ablation of Nrp1 in endothelial cells leads to brain vascular pathologies that are strikingly similar to those that develop in αvβ8 integrin and TGFβ receptor mutant mice [24, 25]. The vascular phenotypes in Nrp1 mutants are not due to defective semaphorin binding and genetic ablation of VEGF-A in the developing CNS does not generate vascular defects that develop in Nrp1 mutant mice [38]. Hence, the reactivation of the Nrp1-TGFβ pathway in GBM cells to promote tumor progression is a likely pathological recapitulation of events involved in brain vascular development.

## Supporting information

S1 Fig NRP1 and NRP2 gene expression analysis in human GBM tissues. **(A);** Analysis of GBM TCGA database reveal that NRP2 RNA expression levels are not statistically different in GBM versus normal brain or lower grade brain tumors. **(B);** NRP2 is enriched in mesenchymal GBM samples based on analysis of the GBM TCGA database. **(C);** Comparative expression of NRP1 and NRP2 in various tumor regions based on IVY GBM database queries. **(D);** Overall survival plot taken from TCGA dataset for patients with primary GBM. Patients were segregated based on high levels of NRP1 expression (n = 195) versus low levels of NRP1 expression (n = 153). Note that high versus low levels of NRP1 mRNA do not lead to statistical differences in overall patient survival.
(JPG)

S2 Fig Confirmation of Nrp1 antibody immunohistochemical specificity in human GBM samples. **(A-D);** Immunohistochemistry stains of two different GBM samples using control IgG (A, C) an anti-Nrp1 antibody (B, D) reveals specificity of the anti-Nrp1 antibody.
(JPG)

S3 Fig Analysis of Nrp1 protein expression in the normal human brain. **(A-C);** Formalin fixed paraffin embedded sections through cerebral cortices of the human fetal brain were immunohistochemically labeled with anti-Nrp1 antibodies (A, B) or control IgG (C). Note that Nrp1 protein is expressed mainly in intracerebral blood vessels (arrows) in the developing human brain. Scales bars, 50 μm.
(JPG)

S4 Fig Analysis of Nrp1-dependent GBM cell growth in vitro and in vivo. **(A);** Nrp1-dependent proliferation was quantified in cells expressing control (NT) shRNAs or Nrp1 shRNAs by counting cell number every 24 hours over 4 days. Note that silencing Nrp1 expression does not impact LN229 cell proliferation in vitro. **(B-E);** Intracranial implantation on LN229 cells reveals a striking Nrp1-dependent difference in GBM cell growth. Shown are representative images, revealing that Nrp1 silencing leads to more robust tumor cell growth as revealed by H&E staining coronal brain sections. Note the hemorrhage within the tumors derived from Nrp1 shRNA cells (arrows). Panels D, E are higher magnification images of boxed areas in B, C. **(F);** Quantitation of Nrp1-dependent GBM growth in vivo, revealing that LN229 cells expressing Nrp1 shRNAs generate intracranial tumors that are nearly twice as large as control tumors. Error bars represent standard deviation, ***p<0.001 for Nrp1 shRNA versus control shRNA. **(G, H);** Analysis of Nrp1-dependent proliferation as determined by double immunofluorescence with anti-vimentin to label GBM cells (green) and anti-pS10 Histone H3 to identify mitotic cells (red) in control and Nrp1 shRNA orthotopic brain tumors. **(I);** Quantitation of Nrp1-dependent GBM cell proliferation as determined by counting vimentin-expressing tumor cells that are also immunoreactive for pS10 Histone H3. For these experiments we analyzed 5 randomly selected fields in tumors expressing control shRNAs or Nrp1 shRNAs. There are no statistically significant Nrp1-dependent differences in tumor cell proliferation.
(JPG)

S5 Fig Immunofluorescence analysis of Nrp1-dependent GBM cell growth in vivo. **(A-D);** Margins of intracranial tumors formed from LN229 cells expressing control shRNAs or shRNAs targeting Nrp1 were labeled with antibodies recognizing human vimentin to visualize tumor cells and GFAP to visualize astrocytes (A, B). Alternatively tumor sections were labeled with anti-vimentin to image tumor cells in combination with anti-Iba1 to visualize astrocytes and microglial cells (C, D).
(JPG)

S6 Fig Analysis of Nrp1-dependent GSC growth in vitro and in vivo. **(A);** Anti-Nrp1 immunoblot of six different primary GSC cultures reveals varying levels of Nrp1 protein expression. **(B);** Lentivirus expressing non-targeting control shRNAs or Nrp1 shRNAs were used to silence Nrp1 expression in GSC7-2 cells, as revealed by anti-Nrp1 immunoblots. **(C);** Images of GSCs expressing GFP in combination with control shRNAs or Nrp1 shRNAs. **(D);** GSC proliferation assay results using the Alamar Blue reagent reveals no Nrp1-dependent growth differences in GSCs. **(E, F);** Images of mouse brains harboring tumors generated from GSC7-2 cells expressing control shRNAs (D) or shRNAs targeting Nrp1 (E), imaged by bright field microscopy (top) or with GFP fluorescence (bottom). **(G);** Nrp1-dependent brain tumor volumes were quantified by measuring GFP fluorescence intensity in coronal slices from tumors derived from GSC7-2 expressing control shRNAs (n = 3) or Nrp1 shRNAs (n = 3), *p<0.05 for Nrp1 shRNA versus control shRNA.
(JPG)

S7 Fig Analysis of Nrp1-dependent TGFβ signaling in LN229 GBM cells and HEK-293T cells. **(A);** LN229 cells expressing control shRNAs or shRNAs targeting Nrp1 were stimulated with TGFβ1 for varying times, and Smad3 phosphorylation was analyzed by immunoblotting. **(B);** Quantitation of Nrp1-dependent canonical TGFβ signaling based on one representative immunoblot. Note that RNAi-mediated silencing of Nrp1 leads to reduced Smad3 phosphorylation in response to TGFβ1. **(C);** Detergent-soluble lysates from non-transfected HEK-293T cells were treated with 5 ng/ml TGFβ1 for varying times. Detergent-soluble lysates were immunoblotted with anti-Nrp1, anti-pSmad2 and anti-pSmad3 antibodies. **(D);** HEK-293T cells transiently transfected with a pcDNA3.1 plasmid to overexpress Nrp1 and then stimulated with TGFβ1 for varying times. Detergent-soluble lysates were immunoblotted with anti-Nrp1, anti-pSmad2 and anti-pSmad3 antibodies. Note the time-dependent increased levels of Smad2 and Smad3 phosphorylation after Nrp1 overexpression.
(JPG)

S8 Fig Forced expression of Nrp1 in GBM cells increases Smad3 phosphorylation and enhances TGFβ receptor internalization. **(A);** LN229 GBM cells forcibly expressing Cherry (top) or a Nrp1-Cherry fusion protein (bottom) were treated with 5 ng/ml TGFβ1 for varying times. Cell surface levels of TGFβR2 were analyzed by labeling fixed, non-permeabilized cells by immunofluorescence. **(B);** Quantitation of Nrp1-dependent TGFβR2 cell surface protein levels following TGFβ1 treatment, **p<0.01 for time 0 versus 15 and 30 minutes for Cherry control and **p<0.001 for time 0 versus 5, 15 and 30 minutes for Nrp1-Cherry. **(C);** Detergent-soluble lysates from LN229 cells expressing control Cherry or Nrp1-Cherry fusion protein were analyzed by immunoblotting. Cells were stimulated TGFβ1 for varying times, and Smad3 phosphorylation was analyzed by immunoblotting. **(D);** Quantitation of Nrp1-dependent TGFβ signaling via Smad3. Note that forcibly expressing Nrp1 leads to increased enhanced signaling via Smad3.
(JPG)

S9 Fig Analysis of Nrp1-dependent TGFβ signaling and TGFβR2 internalization in HUVECs. **(A);** Detergent-soluble lysates from HUVECs expressing control shRNAs or Nrp1 shRNAs were immunoblotted with Nrp1 antibodies, revealing diminished Nrp1 expression following RNAi-mediated silencing. **(B);** HUVECs were stimulated with 5 ng/ml TGFβ1 for varying times and detergent-soluble lysates were immunoblotted with anti-pSmad3 antibodies. Note the increased levels of pSmad3 in the absence of Nrp1. **(C);** HUVECs were treated with TGFβ1 for varying times and TGFβR2 internalization was quantified by immunofluorescence. Note that TGFβ1 does not induce time-dependent TGFβR2 internalization. **(D);** HUVECs were labeled with Phalloidin-Alexa594, revealing Nrp1-dependent F-actin cytoskeletal defects.
(JPG)
