# Supplementary figures and images for: Neuropilin-1 modulates TGFβ signaling to drive glioblastoma growth and recurrence after anti-angiogenic therapy
Source: PLoS One. 2017 Sep 22;12(9):e0185065. doi: 10.1371/journal.pone.0185065 (PMC5609745; doi:10.1371/journal.pone.0185065)

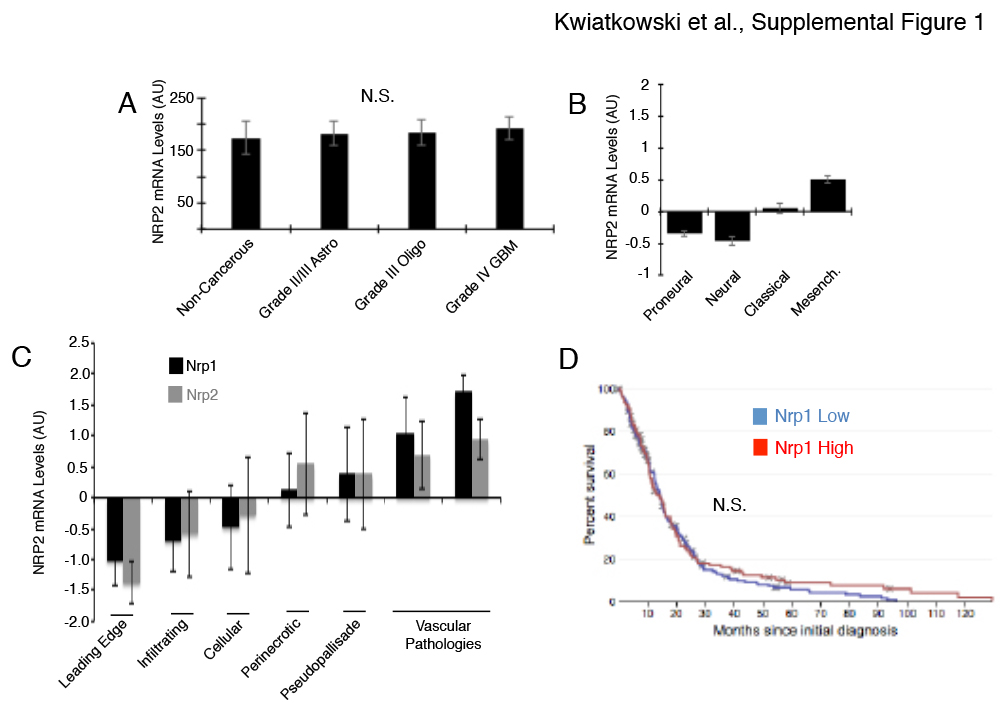

Supplement: S1 Fig — (A); Analysis of GBM TCGA database reveal that NRP2 RNA expression levels are not statistically different in GBM versus normal brain or lower grade brain tumors. (B); NRP2 is enriched in mesenchymal GBM samples based on analysis of the GBM TCGA database. (C); Comparative expression of NRP1 and NRP2 in various tumor regions based on IVY GBM database queries. (D); Overall survival plot taken from TCGA dataset for patients with primary GBM. Patients were segregated based on high levels of NRP1 expression (n = 195) versus low levels of NRP1 expression (n = 153). Note that high versus low levels of NRP1 mRNA do not lead to statistical differences in overall patient survival. (JPG) [file pone.0185065.s001.jpg]

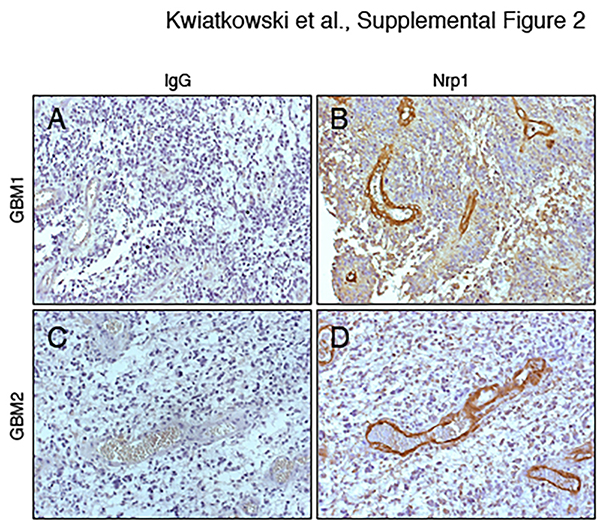

Supplement: S2 Fig — (A-D); Immunohistochemistry stains of two different GBM samples using control IgG (A, C) an anti-Nrp1 antibody (B, D) reveals specificity of the anti-Nrp1 antibody. (JPG) [file pone.0185065.s002.jpg]

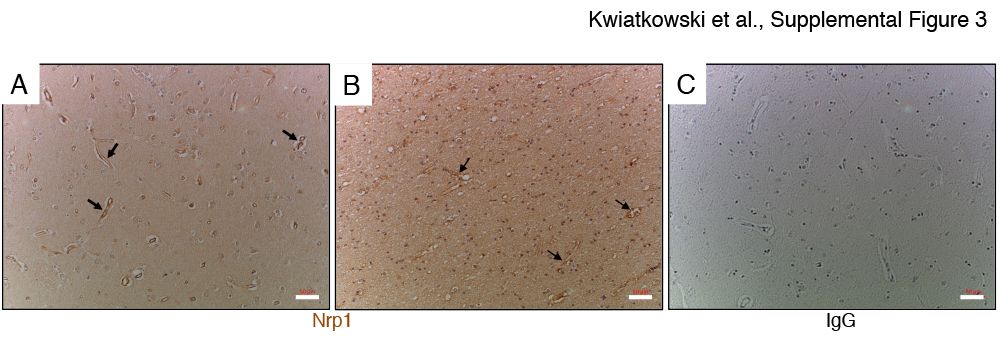

Supplement: S3 Fig — (A-C); Formalin fixed paraffin embedded sections through cerebral cortices of the human fetal brain were immunohistochemically labeled with anti-Nrp1 antibodies (A, B) or control IgG (C). Note that Nrp1 protein is expressed mainly in intracerebral blood vessels (arrows) in the developing human brain. Scales bars, 50 μm. (JPG) [file pone.0185065.s003.jpg]

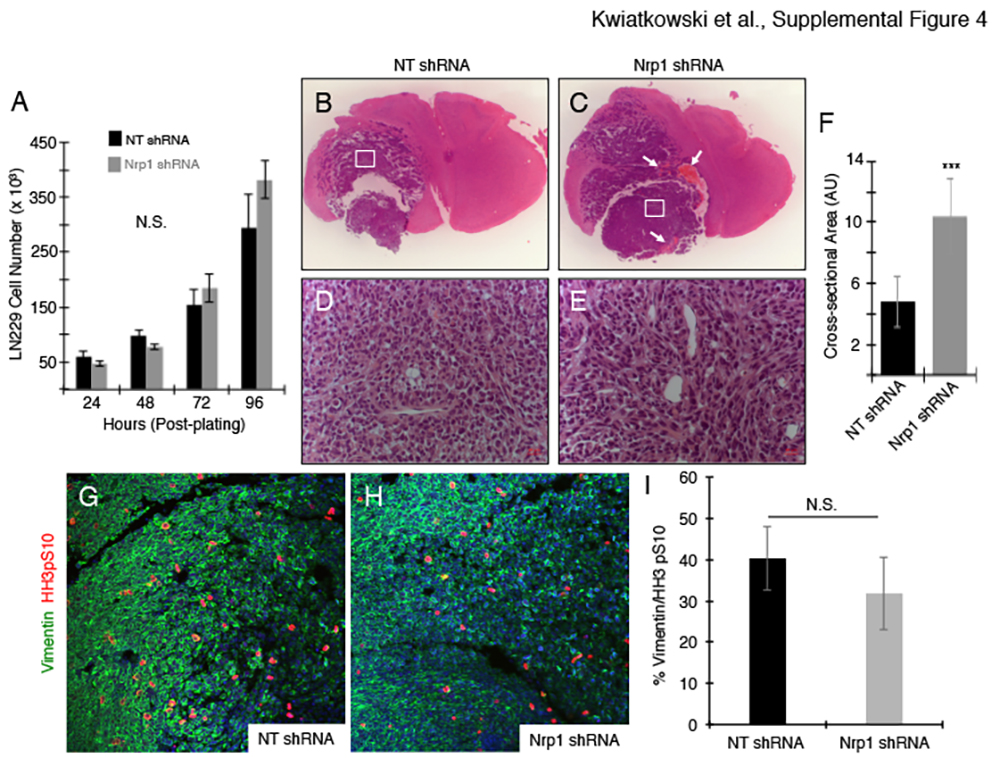

Supplement: S4 Fig — (A); Nrp1-dependent proliferation was quantified in cells expressing control (NT) shRNAs or Nrp1 shRNAs by counting cell number every 24 hours over 4 days. Note that silencing Nrp1 expression does not impact LN229 cell proliferation in vitro. (B-E); Intracranial implantation on LN229 cells reveals a striking Nrp1-dependent difference in GBM cell growth. Shown are representative images, revealing that Nrp1 silencing leads to more robust tumor cell growth as revealed by H&E staining coronal brain sections. Note the hemorrhage within the tumors derived from Nrp1 shRNA cells (arrows). Panels D, E are higher magnification images of boxed areas in B, C. (F); Quantitation of Nrp1-dependent GBM growth in vivo, revealing that LN229 cells expressing Nrp1 shRNAs generate intracranial tumors that are nearly twice as large as control tumors. Error bars represent standard deviation, ***p<0.001 for Nrp1 shRNA versus control shRNA. (G, H); Analysis of Nrp1-dependent proliferation as determined by double immunofluorescence with anti-vimentin to label GBM cells (green) and anti-pS10 Histone H3 to identify mitotic cells (red) in control and Nrp1 shRNA orthotopic brain tumors. (I); Quantitation of Nrp1-dependent GBM cell proliferation as determined by counting vimentin-expressing tumor cells that are also immunoreactive for pS10 Histone H3. For these experiments we analyzed 5 randomly selected fields in tumors expressing control shRNAs or Nrp1 shRNAs. There are no statistically significant Nrp1-dependent differences in tumor cell proliferation. (JPG) [file pone.0185065.s004.jpg]

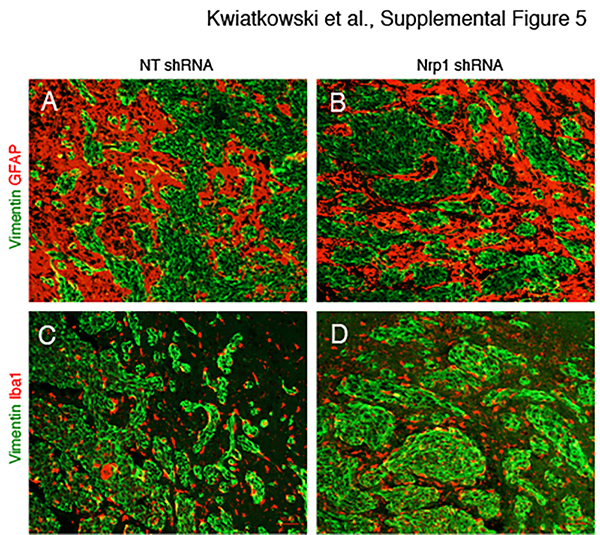

Supplement: S5 Fig — (A-D); Margins of intracranial tumors formed from LN229 cells expressing control shRNAs or shRNAs targeting Nrp1 were labeled with antibodies recognizing human vimentin to visualize tumor cells and GFAP to visualize astrocytes (A, B). Alternatively tumor sections were labeled with anti-vimentin to image tumor cells in combination with anti-Iba1 to visualize astrocytes and microglial cells (C, D). (JPG) [file pone.0185065.s005.jpg]

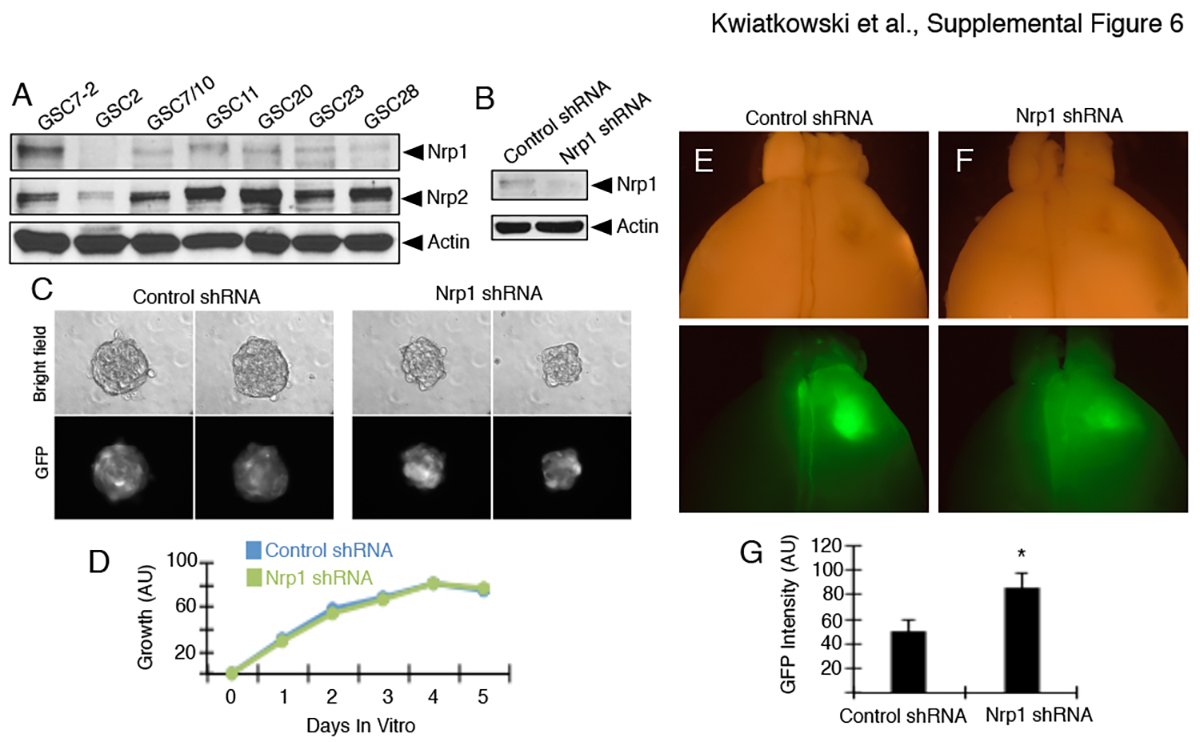

Supplement: S6 Fig — (A); Anti-Nrp1 immunoblot of six different primary GSC cultures reveals varying levels of Nrp1 protein expression. (B); Lentivirus expressing non-targeting control shRNAs or Nrp1 shRNAs were used to silence Nrp1 expression in GSC7-2 cells, as revealed by anti-Nrp1 immunoblots. (C); Images of GSCs expressing GFP in combination with control shRNAs or Nrp1 shRNAs. (D); GSC proliferation assay results using the Alamar Blue reagent reveals no Nrp1-dependent growth differences in GSCs. (E, F); Images of mouse brains harboring tumors generated from GSC7-2 cells expressing control shRNAs (D) or shRNAs targeting Nrp1 (E), imaged by bright field microscopy (top) or with GFP fluorescence (bottom). (G); Nrp1-dependent brain tumor volumes were quantified by measuring GFP fluorescence intensity in coronal slices from tumors derived from GSC7-2 expressing control shRNAs (n = 3) or Nrp1 shRNAs (n = 3), *p<0.05 for Nrp1 shRNA versus control shRNA. (JPG) [file pone.0185065.s006.jpg]

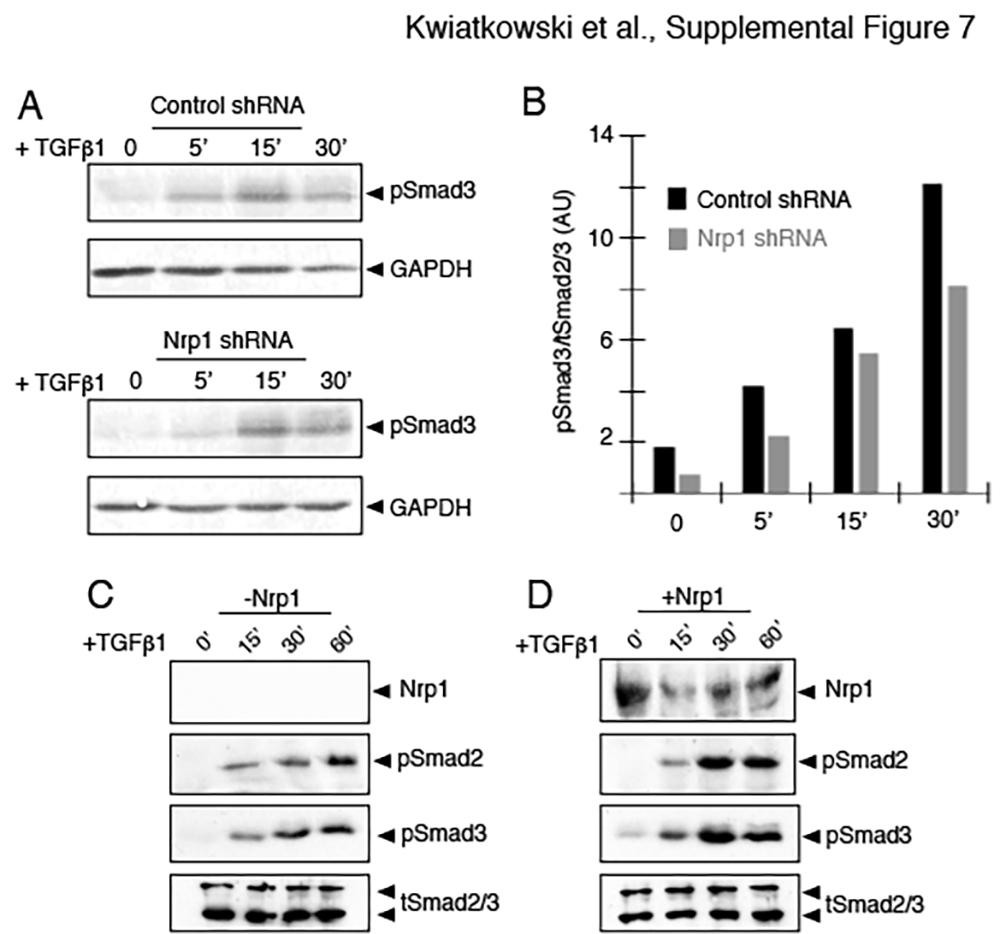

Supplement: S7 Fig — (A); LN229 cells expressing control shRNAs or shRNAs targeting Nrp1 were stimulated with TGFβ1 for varying times, and Smad3 phosphorylation was analyzed by immunoblotting. (B); Quantitation of Nrp1-dependent canonical TGFβ signaling based on one representative immunoblot. Note that RNAi-mediated silencing of Nrp1 leads to reduced Smad3 phosphorylation in response to TGFβ1. (C); Detergent-soluble lysates from non-transfected HEK-293T cells were treated with 5 ng/ml TGFβ1 for varying times. Detergent-soluble lysates were immunoblotted with anti-Nrp1, anti-pSmad2 and anti-pSmad3 antibodies. (D); HEK-293T cells transiently transfected with a pcDNA3.1 plasmid to overexpress Nrp1 and then stimulated with TGFβ1 for varying times. Detergent-soluble lysates were immunoblotted with anti-Nrp1, anti-pSmad2 and anti-pSmad3 antibodies. Note the time-dependent increased levels of Smad2 and Smad3 phosphorylation after Nrp1 overexpression. (JPG) [file pone.0185065.s007.jpg]

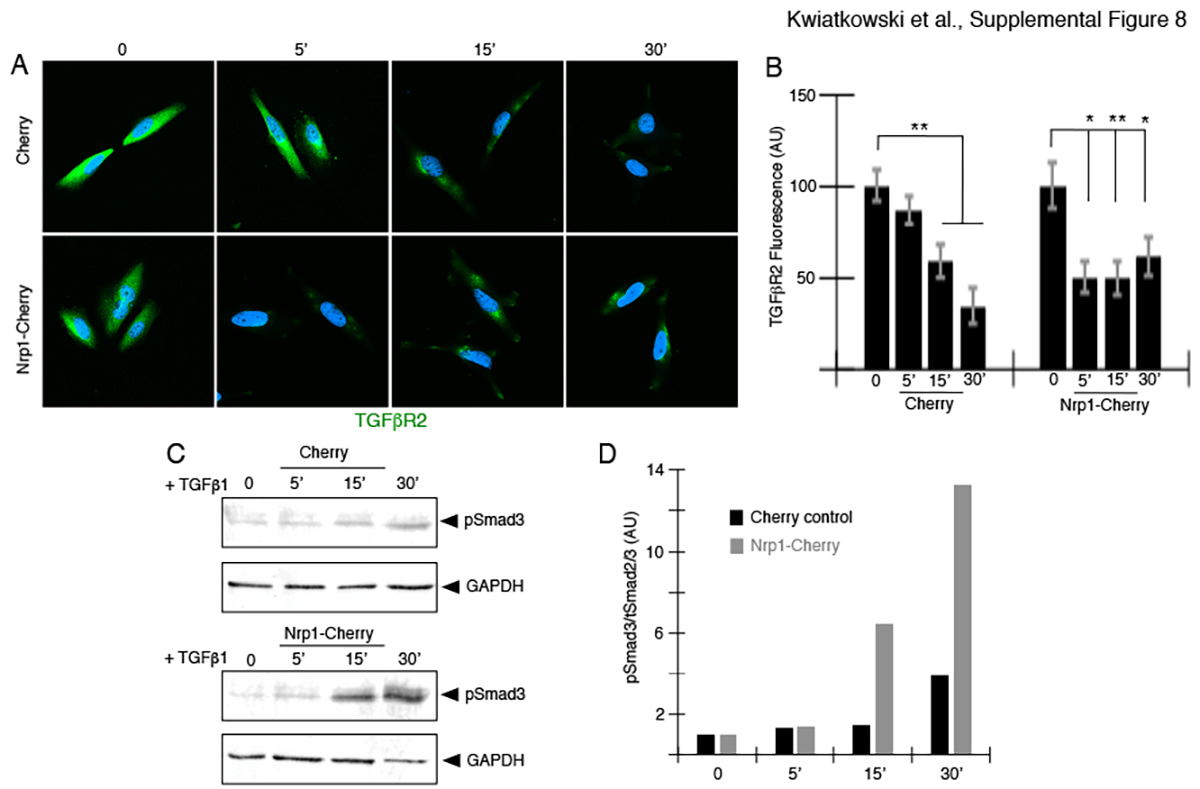

Supplement: S8 Fig — (A); LN229 GBM cells forcibly expressing Cherry (top) or a Nrp1-Cherry fusion protein (bottom) were treated with 5 ng/ml TGFβ1 for varying times. Cell surface levels of TGFβR2 were analyzed by labeling fixed, non-permeabilized cells by immunofluorescence. (B); Quantitation of Nrp1-dependent TGFβR2 cell surface protein levels following TGFβ1 treatment, **p<0.01 for time 0 versus 15 and 30 minutes for Cherry control and **p<0.001 for time 0 versus 5, 15 and 30 minutes for Nrp1-Cherry. (C); Detergent-soluble lysates from LN229 cells expressing control Cherry or Nrp1-Cherry fusion protein were analyzed by immunoblotting. Cells were stimulated TGFβ1 for varying times, and Smad3 phosphorylation was analyzed by immunoblotting. (D); Quantitation of Nrp1-dependent TGFβ signaling via Smad3. Note that forcibly expressing Nrp1 leads to increased enhanced signaling via Smad3. (JPG) [file pone.0185065.s008.jpg]

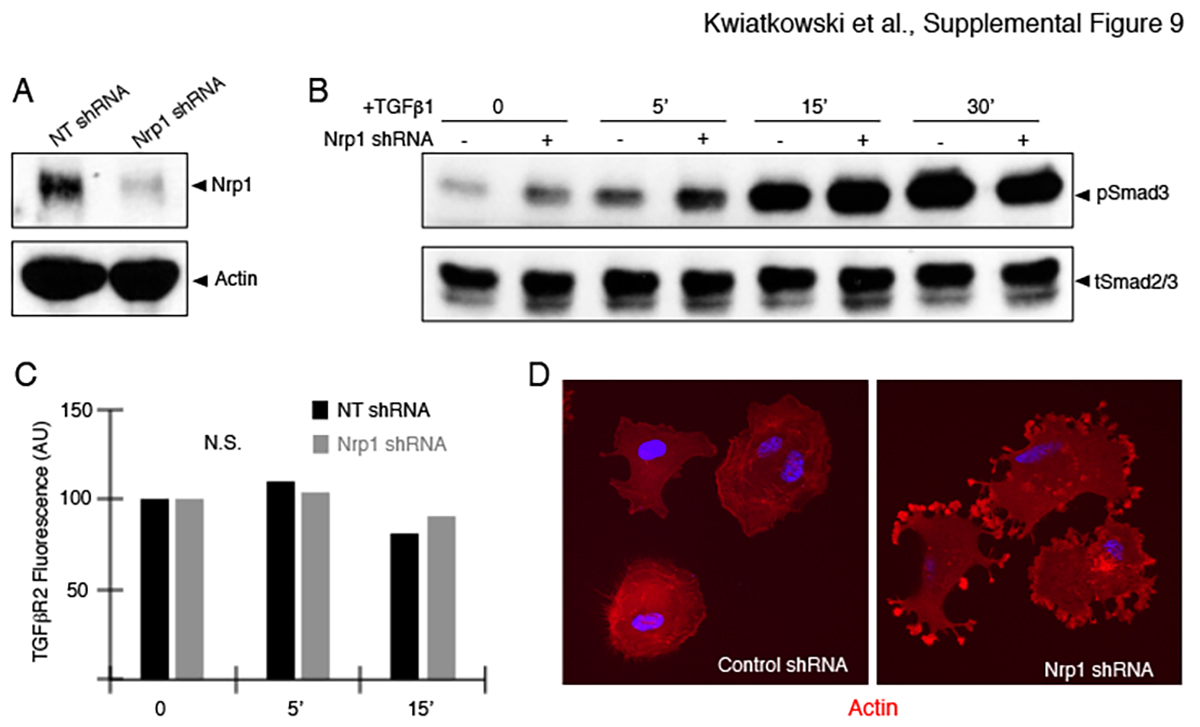

Supplement: S9 Fig — (A); Detergent-soluble lysates from HUVECs expressing control shRNAs or Nrp1 shRNAs were immunoblotted with Nrp1 antibodies, revealing diminished Nrp1 expression following RNAi-mediated silencing. (B); HUVECs were stimulated with 5 ng/ml TGFβ1 for varying times and detergent-soluble lysates were immunoblotted with anti-pSmad3 antibodies. Note the increased levels of pSmad3 in the absence of Nrp1. (C); HUVECs were treated with TGFβ1 for varying times and TGFβR2 internalization was quantified by immunofluorescence. Note that TGFβ1 does not induce time-dependent TGFβR2 internalization. (D); HUVECs were labeled with Phalloidin-Alexa594, revealing Nrp1-dependent F-actin cytoskeletal defects. (JPG) [file pone.0185065.s009.jpg]
